# Pattern of drug utilization for treatment of uncomplicated malaria in urban Ghana following national treatment policy change to artemisinin-combination therapy

**DOI:** 10.1186/1475-2875-8-2

**Published:** 2009-01-05

**Authors:** Alexander NO Dodoo, Carole Fogg, Alex Asiimwe, Edmund T Nartey, Augustina Kodua, Ofori Tenkorang, David Ofori-Adjei

**Affiliations:** 1 Centre for Tropical Clinical Pharmacology & Therapeutics, University of Ghana Medical School, P.O. Box KB 4236, Accra, Ghana; 2 Drug Safety Research Unit, Bursledon Hall, Southampton, SO31 1AA, UK; 3 University of Portsmouth, Portsmouth, UK

## Abstract

**Background:**

Change of first-line treatment of uncomplicated malaria to artemisinin-combination therapy (ACT) is widespread in Africa. To expand knowledge of safety profiles of ACT, pharmacovigilance activities are included in the implementation process of therapy changes. Ghana implemented first-line therapy of artesunate-amodiaquine in 2005. Drug utilization data is an important component of determining drug safety, and this paper describes how anti-malarials were prescribed within a prospective pharmacovigilance study in Ghana following anti-malarial treatment policy change.

**Methods:**

Patients with diagnosis of uncomplicated malaria were recruited from pharmacies of health facilities throughout Accra in a cohort-event monitoring study. The main drug utilization outcomes were the relation of patient age, gender, type of facility attended, mode of diagnosis and concomitant treatments to the anti-malarial regimen prescribed. Logistic regression was used to predict prescription of nationally recommended first-line therapy and concomitant prescription of antibiotics.

**Results:**

The cohort comprised 2,831 patients. Curative regimens containing an artemisinin derivative were given to 90.8% (n = 2,574) of patients, although 33% (n = 936) of patients received an artemisinin-based monotherapy. Predictors of first-line therapy were laboratory-confirmed diagnosis, age >5 years, and attending a government facility. Analgesics and antibiotics were the most commonly prescribed concomitant medications, with a median of two co-prescriptions per patient (range 1–9). Patients above 12 years were significantly less likely to have antibiotics co-prescribed than patients under five years; those prescribed non-artemisinin monotherapies were more likely to receive antibiotics. A dihydroartemisinin-amodiaquine combination was the most used therapy for children under five years of age (29.0%, n = 177).

**Conclusion:**

This study shows that though first-line therapy recommendations may change, clinical practice may still be affected by factors other than the decision or ability to diagnose malaria. Age, diagnostic confirmation and suspected concurrent conditions lead to benefit:risk assessments for individual patients by clinicians as to which anti-malarial treatment to prescribe. This has implications for adherence to policy changes aiming to implement effective use of ACT. These results should inform education of health professionals and rational drug use policies to reduce poly-pharmacy, and also suggest a potential positive impact of increased access to testing for malaria both within health facilities and in homes.

## Background

Artemisinin combination therapy (ACT) is becoming first-line treatment for uncomplicated *Plasmodium falciparum *malaria episodes throughout Africa. The urgency for ACT roll-out was spurred by alarming levels of drug resistance to previously used monotherapies such as chloroquine and sulphadoxine-pyrimethamine (SP) and rising morbidity and mortality [1,2]. Kwa-Zulu Natal was the first health ministry to switch to an ACT in 2001 [3], and ACT have now been adopted in 28 African countries, with an additional 13 in the process of implementation [4]. In Ghana, the Ministry of Health (MOH) policy changed, in 2004, in line with World Health Organization (WHO) recommendations to artesunate plus amodiaquine (ART-AQ) as first-line therapy for uncomplicated malaria from 1^st ^January 2005 [5]. Reports of adverse events with ART-AQ received by the then National Centre for Pharmacovigilance at the University of Ghana Medical School (UGMS) and increasing negative media coverage on the safety of ART-AQ led to withdrawal of specified brands of ART-AQ in December 2005 [6]. This was accompanied by a fall in the number of reports though there had been 131 reports of adverse events (AEs) to ART-AQ to the UGMS by October 2007 [7].

Active post-marketing drug safety surveillance using prescription-event monitoring methodology is practiced both in the UK [8] and New Zealand [9] by collecting event information for patients prescribed new drugs to detect previously unknown drug effects or to better characterize known effects in 'real-life'. Within the context of post-marketing monitoring for adverse events, a knowledge of the patterns of drug utilization, both in terms of prescriber characteristics and patient population, are essential to characterize the potential risks that patients may experience. In March 2006, a non-interventional pharmacovigilance study was initiated in Accra to complement the spontaneous reporting system for newly introduced anti-malarials. The study was originally designed to compare the 'real-life' safety profiles of ART-AQ and chlorproguanil-dapsone (LapDap™) as they are used in real-life settings in Ghana. However, due to the limited use of LapDap™ following introduction of ACT [10] and the progress of the development of chlorproguanil-dapsone-artesunate [11], data on the existing available anti-malarial regimens was collected though the concerns above caused the study to be formally terminated by the sponsor, WHO-TDR, in November 2006.

The objective of this paper is to describe the mode of diagnosis and pattern of drug management in outpatients diagnosed with suspected uncomplicated malaria in health facilities in Accra, Ghana who were recruited into a prospective pharmacovigilance study. Safety analysis of adverse events experienced during treatment with the anti-malarials reported in the cohort will be presented in a forthcoming paper.

## Methods

Data presented in this article were collected during a prospective, longitudinal, non-interventional study to monitor adverse events (AEs) following administration of anti-malarials for uncomplicated malaria in Accra, the capital city of Ghana.

### Study design and sample size

A cohort-event monitoring methodology was used, in which all new patients prescribed anti-malarials for uncomplicated malaria, in the normal course of medical practice in the participating healthcare facilities in Accra were eligible for recruitment; patients with severe/complicated malaria and pregnant women were excluded. An urban area was chosen to be able to recruit a large cohort in a short time period, and to reduce logistical constraints of follow-up as many people were contactable by telephone and lived nearby to facilitate home visits. The variety of health facilities and large numbers of health professionals enabled assessment of a wide range of practices. The average prevalence of malaria in children in Accra is estimated at 14.8%, ranging from 6% to 22% by community [12]. The sample size calculation for the original study was based on the possibility of detecting a rare adverse event at a frequency of 1 in 3,000 patients – that is a sample size of 10,000 patients for each drug [13]. Although the study was discontinued prematurely by the sponsor, a substantial number of patients had already been included in the cohort.

### Recruitment

All public health facilities in Accra were approached to participate in the study and agreed to take part. Private facilities with adequate staff availability and patient load were also invited to participate. The 24 health institutions who participated are thought to be representative of practice across Accra; 15 government hospitals/clinics, two quasi-government hospitals, one private clinic and six community pharmacies. Each facility was asked to sign a consent form to indicate acceptance for participation. All facilities operate a 'fee for service' system and fees are very similar across facility types. Eligible patients were recruited consecutively from the pharmacy at the time of dispensing the drug in order to reduce the number of patients who might be prescribed anti-malarials but who may be unable to obtain their supplies either due to availability of medicines or lack of patient funds. Patient recruitment rates varied based on staff time and the ability of research team to post dedicated data collectors to the sites.

### Data collection

Data collected on the day of anti-malarial dispensing included demographics, contact details, method of diagnosis (health professional presumptive diagnosis [by type of health professional] or slide-confirmed), anti-malarial drugs prescribed and concomitant medications dispensed on that day. All recruited patients were given a study card which they could present at the Out-Patients Department of the Korle-Bu Polyclinic (affiliated to the teaching hospital) in case their clinical condition did not improve and they were unable or unwilling to visit a primary care facility or in case of adverse events for which the patient wished to seek hospital treatment. Collection of adverse event data by systematic follow-up contact by the research team and use of spontaneous report forms by patients will be discussed in a subsequent paper.

### Data management and analysis

Each enrolled patient was given a unique study number that was used in the storage and management of all data relating to the patient. Data were double entered, data cleaned and managed using Microsoft Access (Microsoft Corporation, Redmond, Washington) and analysed using STATA version 9.2, (Stata Corporation, College Station, Texas). Anti-malarial treatment groups were defined as follows: i) recommended first-line therapy (artesunate plus amodiaquine – ART-AQ); ii) 'other ACT' group – patients taking an artemisinin derivative in combination with a non-artemisinin-containing anti-malarial; iii) monotherapy (artemisinin) group – patients taking only artemisinin-based compounds for treatment of malaria episode, including more than one type of artemisinin e.g. artemether injection followed by oral artesunate; iv) monotherapy (other) group – other monotherapy regimens not containing artemisinin compounds. Data was expressed as means and standard deviations (SD) for continuous variables, and frequencies and percentages for categorical variables. Categories of the variable 'facility type' were regrouped into government facilities vs. non-government facilities (quasi-government, private clinic, community pharmacy) due to small numbers and common characteristics within the non-government group. Categories of the variable 'mode of diagnosis' were regrouped into laboratory confirmed diagnosis vs. presumptive diagnosis (including doctor, pharmacist, other health professional and self) categories. Chi-square and Fishers' Exact Test were used for categorical variables and students T-test for continuous variables for univariate analysis to predict prescription of: i) first-line therapy (ART-AQ); ii) antibiotics. Results were considered statistically significant at a level of p < 0.05.

A stepwise multivariate logistic regression method was used to assess the effect of variables on the outcomes of prescription of first line therapy and antibiotics using a significance level for entry of the variable/category into the model of p = 0.15. The performance of the model was assessed on calibration and discrimination using Hosmer-Lemeshow goodness-of-fit test and area under the Receiver Operating Characteristic (ROC) curve respectively. 'Discrimination' refers to a variable's ability to distinguish prescription of first line therapy/antibiotic use from no prescription of first line therapy/no antibiotic use. The AUC represents the probability that a patient who had ART-AQ or used antibiotics had a higher predicted probability than those who did not. An AUC of 0.5 indicates that the variables do not predict better than chance. The discrimination of the variables is considered perfect if AUC = 1, good if AUC >0.8, moderate if AUC is 0.6 to 0.8, and poor if AUC <0.6. 'Calibration' refers to the agreement between predicted probabilities and the 'true probabilities', which can be approximated by taking the mean of the observed outcomes within predefined groups of patients. The Hosmer-Lemeshow test compares the observed outcome in a group with the predicted outcome of that group [14]. The C goodness-of-fit statistic sorts observations according to their expected probability and partitions the observations into ten groups of equal size. A high H relates to a small *p *value, implying significant difference between observed and predicted outcome, and thus indicates a lack of fit of the variables.

### Ethical approval

Ethical approval for the project was obtained from the Ethical and Protocol Review Committee of the University of Ghana Medical School. The original protocol was also approved by WHO/TDR internal ethical procedures. Participation for patients was subject to a signed and witnessed informed consent form by the patient or his/her carer or guardian for children (under 18 years).

## Results

Recruitment was carried out between April 2006 and November 2006. Of patients who were diagnosed with malaria and attended pharmacies to collect medication, more than 95% consented to participate. The most common reason for non-participation was time constraints for the patient. The final number of patients recruited was 2,835, although four of these were excluded from analysis due to ambiguity of anti-malarial prescribed. The majority of patients were recruited from government facilities (95.9%, n = 2,716).

### Cohort characteristics and malaria diagnosis

60.6% of the cohort was in the 13–59 year age band (Table 1). Although overall females comprised 59.9% of the cohort, the proportion of females increased with increasing age, from 48.4% of under 5's to 69.4% of the over 60's (Chi square trend for those with known age = 63.6, p < 0.001). The major mode of diagnosis of malaria was presumptive diagnosis by a physician (Table 1). Only 3.2% (n = 91) of diagnoses were parasitologically confirmed, with the highest proportion of laboratory confirmed diagnoses in the 5–12 years age group (6.0%, n = 19).

**Table 1:** Characteristics according to age group

	**Age group**					**Total**
	**<5**	**5–12**	**13–59**	**≥60**	**NK**	
	**n = 610** **(21.6%)**	**n = 317** **(11.2%)**	**n = 1,716** **(60.6%)**	**n = 157** **(5.5%)**	**n = 31** **(1.1%)**	**N = 2,831**
	*n (%)*	*n (%)*	*n (%)*	*n (%)*	*n (%)*	*n (%)*
**Gender**						
Male	315 (51.6)	151 (47.6)	606 (35.3)	48 (30.6)	16 (51.6)	1,136 (40.1)
Female	295 (48.4)	166 (52.4)	1,110 (64.7)	109 (69.4)	15 (48.4)	1,695 (59.9)
						
**Facility type**						
Community pharmacy	4 (0.7)	1 (0.3)	18 (1.1)	3 (1.9)	0 (0)	26 (0.9)
Government	599 (98.2)	312 (98.4)	1,621 (94.5)	153 (97.5)	31 (100)	2,716 (95.9)
Private clinic	4 (0.7)	1 (0.3)	27 (1.6)	0 (0)	0 (0)	32 (1.1)
Quasi-government	3 (0.5)	3 (1.0)	50 (2.9)	1 (0.6)	0 (0)	57 (2.0)
						
**Mode of diagnosis**						
Doctor	501 (82.1)	242 (76.4)	1,383 (80.6)	125 (79.6)	18 (58.1)	2,269 (80.2)
Laboratory Confirmation	20 (3.3)	19 (6.0)	48 (2.8)	3 (1.9)	1 (3.2)	91 (3.2)
Other health professional	18 (3.0)	22 (6.9)	127 (7.4)	8 (5.1)	5 (16.1)	180 (6.4)
Pharmacist	0 (0)	0 (0)	10 (0.6)	1 (0.6)	0 (0)	11 (0.4)
Self	0 (0)	2 (0.6)	6 (0.4)	0 (0)	0(0)	8 (0.3)
Unknown	71 (11.6)	32 (10.1)	142 (8.3)	20 (12.7)	7 (22.6)	272 (9.6)
						
**Anti-malarial treatment prescribed**						
Combination therapy (ART-AQ)	161 (26.4)	181 (57.1)	790 (46.0)	76 (48.4)	9 (29.0)	1,217 (43.0)
Combination therapy (Other ACT)	240 (39.3)	40 (12.6)	124 (7.2)	5 (3.2)	9 (29.0)	418 (14.8)
Monotherapy (Artemisinin based)	111 (18.2)	55 (17.4)	700 (40.8)	61 (38.9)	9 (29.0)	936 (33.1)
Monotherapy (Non-artemisinin)	98 (16.1)	41 (12.9)	102 (5.9)	15 (9.6)	4 (12.9)	260 (9.2)

NK = not known

### Anti-malarial treatment prescribed

There were 23 different anti-malarial regimens recorded (Table 2). The majority of patients (90.8%) received an artemisinin-based compound in their treatment regimen. However, only 43.0% received the recommended first line therapy of ART-AQ, although this was the most common regimen reported. Dihydroartemisinin (DHA) was used next most frequently, either alone (20.4%) or combined with another anti-malarial (9.4%). The proportion of patients according to characteristic who were prescribed ART-AQ as opposed to the other regimens is shown in Table 3, with logistic regression results in Table 4. It is of note that of the 91 microscopically confirmed diagnoses, 86.8% (n = 79) were prescribed ART-AQ (adjusted OR 9.7 [5.2–18.2]). Other factors related to ART-AQ prescription were patients aged five years or above and attendance of a government facility. The goodness of fit test for the regression model had a Hosmer-Lemeshow chi square value= 0.07, (2 degrees of freedom), p-value = 0.97. The area under the ROC curve was 0.63 indicating a moderate fit of the model.

**Table 2:** Anti-malarial treatment prescribed

**Drug group/treatment**	**N**	**%**
**1^**st **^line therapy (ART-AQ)**	**1,217**	**43.0**
**Combination therapy – other ACT**	**418**	**14.8**
DHA+AQ	240	8.5
Artemether+AQ	77	2.7
Artemether+lumefantrine	58	2.1
DHA+pyrimethamine-sulphametopirazine	13	0.5
Artesunate+SP	12	0.4
DHA+SP	6	0.2
DHA+chloroquine	5	0.2
Artesunate+chloroquine	3	0.1
Artemether+pyrimethamine-sulphametopirazine	1	0.04
Artemether+quinine	1	0.04
Artesunate+ quinine	1	0.04
DHA+artemether+lumefantrine	1	0.04
**Monotherapy – artemisinin based**	**936**	**33.1**
DHA	577	20.4
Artesunate	293	10.4
Artemether	55	1.9
Artesunate/artemether	10	0.4
Artemether/DHA	1	0.04
**Monotherapy – non-artemisinin**	**260**	**9.2**
Amodiaquine	133	4.7
SP	78	2.8
Pyrimethamine-sulphametopirazine	32	1.1
Chloroquine	14	0.5
Quinine	3	0.1
**Total**	**2,831**	

DHA = dihydroartemisinine; AQ = amodiaquine; SP = sulphadoxine-pyrimethamine

**Table 3:** Characteristics of patients prescribed first-line therapy (ART-AQ) or a co-prescription of antibiotics

	**Anti-malarial prescribed**				**Antibiotic co-prescription**			
	**ART-AQ**		**Other anti-malarial regimen**		**Antibiotic co-prescription**		**No antibiotic**	
	N = 1,217		N = 1,614		N = 872		N = 1,959	
	*n, %*^1^		*n, %*		*n, %*		*n, %*	

**Gender**								
Male	464	40.9	672	59.2	376	33.1	760	66.9
Female	753	44.4	942	55.6	496	29.3	1,199	70.7
								
**Age group**								
<5	161	26.4	449	73.6	260	42.6	350	57.4
5–12	181	56.9	136	43.1	119	37.4	198	62.5
13–59	790	46.0	926	54.0	445	25.9	1, 271	74.1
≥60	76	48.4	81	51.6	40	25.5	117	74.5
*Not known*	*9*	*29.0*	*22*	*71.0*	*8*	*25.8*	*23*	*74.2*
								
**Facility type**								
Non-government	15	13.0	100	87.0	28	24.4	87	75.7
Government	1,202	44.3	1,514	55.7	844	31.1	1,872	68.9
								
**Mode of diagnosis**								
Presumptive	1,041	42.2	1,427	57.8	758	30.7	1,710	69.3
Laboratory confirmed	79	86.8	12	13.2	21	23.1	70	76.9
*Not known*	*97*	*35.7*	*175*	*64.3*	*93*	*34.2*	*179*	*65.8*
								
**Anti-malarial treatment prescribed**								
Combination therapy (ART-AQ)	-		-		349	28.7	868	71.3
Combination therapy (Other ACT)	-		-		140	33.5	278	66.5
Monotherapy (Artemisinin based)	-		-		277	29.6	659	70.4
Monotherapy (Non-artemisinin)	-		-		106	40.8	154	59.2

^1 ^% are row percentages within supercolumns

**Table 4:** Predictors of first-line therapy prescription and co-prescription of concomitant antibiotics^1,2^

	**Prescription of first-line therapy (ART-AQ)**		**Antibiotic use**	
	**Adjusted odds ratio** **[95% CI]**	**p-value**	**Adjusted odds ratio** **[95% CI]**	**p-value**

**Age group**				
<5	1.0		1.0	
5–12	3.6 [2.6–4.9]	<0.001	-	-
13–59	2.5 [2.0–3.1]	<0.001	0.53 [0.44–0.64]	<0.001
≥60	2.6 [1.8–3.9]	<0.001	0.55 [0.37–0.83]	0.004
				
**Facility type**				
Non-government	1.0		-	-
Government	6.3 [3.6–11.1]	<0.001	-	-
				
**Mode of diagnosis**				
Presumptive	1.0		1.0	
Laboratory confirmed	9.7 [5.2–18.2]	<0.001	0.68 [0.41–1.1]	0.13
				
**Anti-malarial treatment prescribed**				
Combination therapy (ART-AQ)	-	-	1.0	
Combination therapy (Other ACT)	-	-	-	-
Monotherapy (Artemisinin based)	-	-	1.2 [0.97–1.4]	0.11
Monotherapy (Non-artemisinin)	-	-	1.4 [1.1–1.9]	0.02

^1 ^Gender was not significant in either of the final models

^2 ^N = 2,535 for both models. Patients with unknown age (n = 31) and/or unknown mode of diagnosis (n = 272) were excluded.

### Co-prescribed medications

Co-prescribed medications were given to 89.5% of the cohort (n = 2,533), with a median number of two per patient (range 1–9). The most commonly prescribed concomitant medications were analgesics, given to 76.3% (n = 2,162) of patients, followed by antibiotics (30.8%, n = 872), and vitamins (25.8%, n = 729). The proportion of patients according to characteristic who were prescribed antibiotics and logistic regression results are in Tables 3 and 4 respectively. Patients aged over 12 years were significantly less likely to have an antibiotic co-prescription than patients under 5 years, while patients prescribed non-artemisinin monotherapies were significantly more likely to have anti-biotic co-prescription. The Hosmer-Lemeshow goodness of fit test had a chi-square of 4.85, (df = 3), p-value = 0.18, indicating good fit. However, the area under the ROC curve was poor with an AUC = 0.58.

### Children under five years

There were 610 patients aged under five years in the cohort and this age group was more likely to be prescribed a DHA-AQ combination (29%, n = 177), rather than ART-AQ (26.4%, n = 161). Amodiaquine and DHA were used as monotherapies in a further 15.7% (n = 96) and 13.8% (n = 84) respectively. Only two children under five years received a non-artemisinin monotherapy (chloroquine). Under-fives were the most likely to receive an antibiotic (42.6%, n = 260), and the least likely to receive an analgesic (60.8%, n = 371) or a vitamin (15.4%, n = 94).

## Discussion

This evaluation, including predominantly government facilities in an urban setting, showed that nearly two years following the change in national anti-malarial policy, the first-line therapy was adhered to in less than 50% of cases. It is possible that the initial safety concerns associated with ART-AQ and availability of a wide variety of alternatives in addition to the fact that patients pay at the point of service delivery could have contributed to this. Artemisinins, either as monotherapy or combination therapy, were used to treat more than 90% of cases of uncomplicated malaria, indicating widespread acceptance of artemisinin therapies and their extensive absorption into urban health facilities. However, 33.1% of patients received an artemisinin monotherapy, a regimen which has not been recommended by WHO since January 2006 due to the potential for drug resistance in this class of drugs due to its short half-life. The reasons for this may include lack of availability of ACTs or safety concerns of the prescriber following the media coverage of side effects of amodiaquine. The need to educate health care workers to stop recommending artemisinin monotherapy is obvious and urgent. This situation could be expected to be different in rural areas, where there is less choice of drugs and where subsidized ART-AQ is available in health centres. The diversity of regimens used has implications for post-marketing safety monitoring, where correct ascertainment of exposure is necessary for attribution of potential adverse effects to a specific suspect drug.

This study illustrated some practical realities of the day-to-day diagnosis and treatment of uncomplicated malaria. Firstly, the high level of presumptive diagnosis of malaria, based on country-specific guidelines [5], seems to be a limiting factor for prescribing the new first-line therapy. A patient with a parasitologically confirmed diagnosis was 9 times more likely than a patient with presumptive diagnosis to be prescribed the first-line therapy, suggesting that the confidence of health professionals in adhering to new guidelines is enhanced by having access to confirmatory tests. Similar findings in Zambia where the first-line therapy artemether-lumefantrine was prescribed more frequently to patients with positive blood smears or rapid diagnostic tests (RDTs) relative to those with a negative test would support this [15]. Overall, only 3.2% of the cohort had a positive test result, indicating very low rates of use of malaria diagnostics. This would support calls to roll-out newer diagnostic technologies such as rapid diagnostic tests (RDTs) for malaria in conjunction with changes to ACT first line therapy to limit over-use of ACTs [16].

Secondly, there was a high level of co-prescription of antibiotics (30.8% of patients receiving at least one antibiotic prescription), which is promoted under febrile illness strategies [17]. The widespread concomitant use of antibiotics has implications for patient safety in increasing the potential for drug-drug interactions, and also for pharmacovigilance, as antibiotics have a high incidence of common adverse events such as rashes and pruritis, which may be ascribed to the new anti-malarials by patients/health care providers. The fact that non-artemisinin monotherapy was significantly more likely to be prescribed with an antibiotic would suggest that these are patients for whom the prescriber is less confident of a true diagnosis of malaria and is prescribing anti-malarials as a 'cover' for potential infection or to prevent subclinical infection becoming manifest. This correlates with findings in Tanzania for patients with a history of cough within the previous 48 hours and a negative blood slide being predictive of no anti-malarial treatment given (as opposed to prescribing anti-malarials even with a negative result) [18]. The benefit:risk decision of the clinician whether or not to co-prescribe could also depend on factors such as presence of measurable fever, ability of the patient to afford the multiple medications, likelihood of the patient being able to return if the condition worsens and previous treatments the patient received.

Although the under-5 age group were the least likely group to receive the recommended ART-AQ regimen (29.0%), this age group seemed to be managed the least conservatively overall, with only 2 patients receiving chloroquine and no sulphonamides prescribed, and a significantly higher proportion of antibiotic prescriptions. Similar findings have been shown in Zambia and Kenya, where 11% and 26% of children were prescribed the recommended ACT shortly after implementation [19,20]. The preference for the DHA-AQ over ART-AQ may reflect clinician expectation of drug efficacy and/or safety in this population more likely to have higher parasitaemias and severe morbidity, the types of paediatric formulations available or the promotional activities of pharmaceutical companies operating in Accra. A qualitative study would be useful to explore this.

There were several potential limitations to this study. Recruitment was potentially biased towards facilities with a high patient load, which were mainly government facilities. However, this usage of facilities reflects the health-seeking behaviour of patients, and is therefore likely to be a representative sample of the population with suspected malaria in Accra. Utilization of government health facilities far outstrips that of private facilities, most of which are small. Other background data on presentation was not collected and this information could have been useful in understanding the treatment regimen prescribed, for example presence of fever on examination, suspected concomitant infections and previous intake of anti-malarials. Drug availability in facilities was not monitored, but stock-outs highly unlikely due to the multiplicity of products available for purchase by all facilities, both public and private. This study was purely non-interventional and did not assess whether prescription was appropriate for age and weight. There was also no information collected on the availability of diagnostics, which may have explained the low utilization, although this is more likely to be due to high patient load and lack of time to wait for the result. Finally, the level of training of staff or familiarity with guidelines was not assessed as a potential factor contributing to the variance in prescribing, but as the majority of facilities were government facilities in the capital city, these staff would have had the highest likelihood of access to training and related resources.

In summary, this study further adds support to the statement that global eradication of malaria, as called for by Bill and Melinda Gates in October 2007 and supported by WHO, the US President's Malaria Initiative and all major players [21], is unlikely unless there is proper diagnosis and management of malaria in all health facilities, including those in resource-constrained environments. This study suggests an important role for confirmatory diagnostics in rational prescribing, which would add support for increased access to diagnostic tools to positively impact on patient management. Continued education of government and private providers on the new national anti-malarial guidelines is also recommended. Knowledge of drug utilization patterns is key in understanding patient management and consequent drug safety issues, and this paper demonstrates the potential difficulties of pharmacovigilance activities for a recommended first-line therapy in an environment in which a wide variety of curative regimens are used. The challenge of pharmacovigilance in environments with limited routine data collection facilities should not be underestimated and requires significant innovation.

## Competing interests

The authors declare that they have no competing interests.

## Authors' contributions

AD and DO-A were the co-investigators for this study and designed the project proposal and oversaw the project. ET was responsible for database design, data entry and pre-analysis management. AK and OT were the research associates implementing the study, managing the data collectors and visiting the various project sites. CF and AA performed statistical analysis. CF and AD drafted the manuscript, and all authors read and approved the final manuscript.
